# From Depletion to Restoration: Lessons From Long‐Term Monitoring of Carbon Gains and Losses in Cropping Systems

**DOI:** 10.1111/gcb.70291

**Published:** 2025-06-09

**Authors:** Caitlin E. Moore, Bethany Blakely, Taylor L. Pederson, Nuria Gomez‐Casanovas, Christy D. Gibson, Anya M. Knecht, Guler Aslan‐Sungur, Evan H. DeLucia, Emily A. Heaton, Andy VanLoocke, Tilden Meyers, Carl J. Bernacchi

**Affiliations:** ^1^ School of Agriculture and Environment The University of Western Australia Crawley Western Australia Australia; ^2^ Centre for Water and Spatial Science The University of Western Australia Crawley Western Australia Australia; ^3^ Center for Advanced Bioenergy and Bioproducts Innovation University of Illinois at Urbana‐Champaign Urbana Illinois USA; ^4^ Department of Atmospheric and Oceanic Sciences University of Wisconsin‐Madison Madison Wisconsin USA; ^5^ Rangeland, Wildlife & Fisheries Management Department Texas A&M College Station Texas USA; ^6^ Texas A&M AgriLife Research Center Texas A&M University Vernon Texas USA; ^7^ Department of Crop Sciences University of Illinois at Urbana‐Champaign Urbana Illinois USA; ^8^ Department of Agronomy Iowa State University Ames Iowa USA; ^9^ Department of Plant Biology University of Illinois at Urbana‐Champaign Urbana Illinois USA; ^10^ NOAA Air Resources Laboratory Boulder Colorado USA; ^11^ United States Department of Agriculture Agricultural Research Service Global Change and Photosynthesis Research Unit Urbana Illinois USA

**Keywords:** carbon accounting, carbon farming, carbon sequestration, climate smart agriculture, cropping systems, eddy covariance, greenhouse gas mitigation, soil organic carbon

## Abstract

As global atmospheric CO_2_ rapidly approaches a key tipping point, there is an urgent need to implement strategies to reverse this pattern. A generally accepted understanding of carbon (C) in agricultural fields includes: (H1) substantial C loss occurs when natural vegetation is converted to crops, (H2) soils typically reach a steady‐state C concentration under contemporary practices, and (H3) improved management or crop selection can enhance soil C stocks over time. Significant variability exists, but studies consistently show large C losses from agricultural ecosystems, supporting H1. Although steady‐state C levels (H2) are commonly assumed, measuring C gains or losses in mature agroecosystems is challenging. Efforts to increase soil C storage (H3) have limited data due to the diversity of potential practices, compounded by substantial variability in soil C measurements. Here, long‐term (7–17 year) ecosystem C flux data from diverse cropping systems revealed that conventionally tilled annual row crops (maize and soybean) act as significant long‐term atmospheric C sources, challenging H2. Furthermore, conservation tillage practices reduced C losses compared with conventional tillage but showed minimal evidence for long‐term ecosystem C storage, even after 20+ years. This indicates that no‐till practices reduce C losses but imply that no soil C is added, challenging H3. By contrast, perennial *Miscanthus × giganteus*, *Panicum virgatum* , and restored tallgrass prairie systems store C at the ecosystem scale more effectively than minimally tilled annual row crops. Analysis over multiple years demonstrates significant ecosystem C storage with perennial crops, varying by species, starting in the first year of transition. These findings, although focused on one region, suggest that the assumptions of steady‐state C levels and increased storage from conservation practices do not universally apply and that significant changes to agroecosystems are required to increase C storage.

## Graphical abstract

As atmospheric CO_2_ continues to rise, enhancing carbon storage in agricultural soils is increasingly important. Traditionally, it is assumed that soils reach stable carbon levels under current practices, and no‐till farming can boost carbon storage. However, long‐term data show that conventionally tilled crops like corn and soybeans continue to release carbon, and even no‐till methods do little to increase storage over decades. In contrast, perennial systems such as switchgrass, miscanthus, and restored prairies do sequester carbon and begin doing so quickly. These findings challenge widely held assumptions of carbon in agroecosystems and highlight the need for bigger changes in agricultural practices.

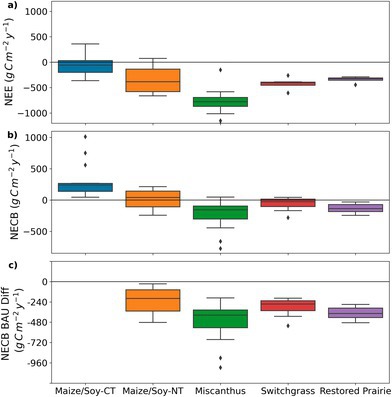

## 1 Introduction

**FIGURE 1:**
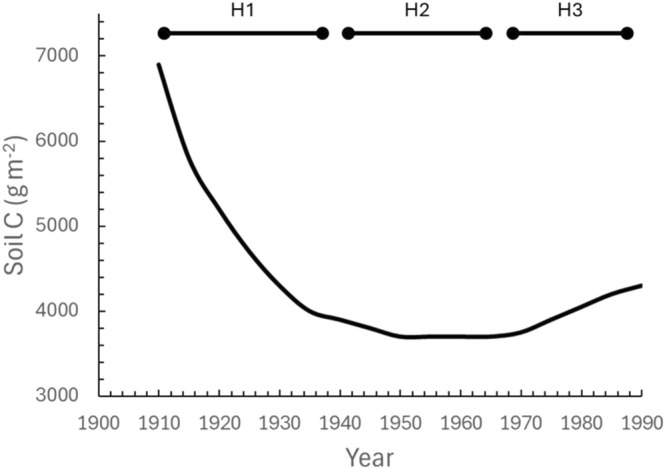
Hypothesized changes in soil C as predicted from Matson et al. (1997). The simulated changes focus on three main hypotheses: (H1) soil carbon losses occurred with the advent of the steel plough that disrupted the prairie soil profile and led to substantial release of stored carbon, (H2) a steady state was reached using conventional agronomic tillage practices, and (H3) tillage practices adopted in the 1970s and onward have increased soil C. Figure modelled after Matson et al. (1997).

Agriculture has experienced a rapid rise in production and intensification since the green revolution of the 1960's and is entering a new age of technological revolution to produce the calories required for global food and energy security (Horton et al. 2021; Wolfert et al. 2017). However, coupled with agricultural production is a greenhouse gas (GHG) emission cost, with land conversion, animal production, and crop management practices, such as tillage, nutrient and pest control additions, and fallow periods leading to an estimated 12% of direct GHG emissions from agricultural land (Masson‐Delmotte et al. 2022). Accounting for the carbon (C) lost during land conversion for agriculture and the following processes that sequester or release it from the soil is an important step needed for balancing GHG inventories (Sanderman and Baldock 2010). Matson et al. (1997) summarised widely accepted hypotheses of C in agricultural ecosystems (Figure 1) as: (H1) a significant amount of C was lost from soils when land was converted from natural vegetation to agricultural ecosystems, (H2) over time a steady‐state soil C concentration is reached with standard agronomic practices, and (H3) that improved management practices or optimal crop species selection can result in increased C storage over time. Although these principles provide useful guidance for improving agroecosystem carbon storage, their validation at commercial scales with long‐term observations remains limited and hinders progress towards determining whether agroecosystems are truly gaining or losing C.

Numerous strategies are being developed to reduce GHG emissions while simultaneously improving agricultural yields, aiming to lessen the environmental impact of food production. In cropping systems, one such approach involves enhancing ecosystem C storage, aligning with H3 specifically, through modifications to management practices (till vs. no‐till), crop selection (annual vs. perennial) and crop rotation regimes (simple vs. diverse). The establishment of voluntary C markets and other C crediting schemes to incentivize more widespread adoption has led to the need for farmers to rapidly apply these strategies (Chabbi et al. 2017; Fujii et al. 2024; Mitchell et al. 2024; Oldfield et al. 2022; Rumpel et al. 2020). Yet, standardized and observation‐based analyses for measuring GHG emissions and ecosystem C storage are lacking in agricultural systems (Ellis and Paustian 2024), which is concerning given the spatial and temporal variability reported in the scientific literature on ecosystem C storage and on how long it can be maintained (Edlinger et al. 2023; Mitchell et al. 2024; Moore et al. 2022; Oldfield et al. 2024; Smith et al. 2020).

Although empirical data from direct soil measurements have been collected for several agroecosystems and management practices, the results from these analyses are often limited in scope or inconclusive due to the challenges presented by the soil environment (e.g., Paustian et al. 2019). Direct measurements of the C pool in soils are prone to limited depth of measurements, highly sensitive to soil texture, soil bulk density changes, subjected to a low signal‐to‐noise ratio, and confounded with substantial spatial heterogeneity, even within the same field plots (Mitchell et al. 2024; Poeplau et al. 2024). Because of this, inferring changes in ecosystem C storage requires both extensive spatial sampling at each time point and long‐term measurements to minimize the signal‐toto‐noise ratio (Bradford et al. 2023; Potash et al. 2025).

Quantifying the impact of conventional versus conservation practices on ecosystem C budgets, however, lacks the spatial scales and duration necessary to resolve changes in ecosystem C storage through direct measurements. An alternate method to direct soil measurements is to quantify fluxes of C into and out of the ecosystem using the eddy covariance technique (e.g., Hollinger et al. 2005; Verma et al. 2005; Zeri et al. 2011). Integrating instantaneous C flux measurements, referred to as net ecosystem exchange (NEE; Chapin et al. 2006) over time with this approach quantifies whether the ecosystem is gaining (i.e., −NEE), losing C (i.e., +NEE), or is C neutral (i.e., NEE ≈ 0). This integration of NEE is defined as Net Ecosystem Productivity (NEP; Chapin et al. 2006). As agricultural ecosystems are managed to produce crop yields, the C harvested represents a significant fraction of NEP. Accounting for this C harvest by removing it from NEP reflects the amount of C change in the ecosystem, defined as net ecosystem carbon balance (NECB; Chapin et al. 2006). Although there are other potential fates of C entering and leaving agroecosystems, these are generally considered to be negligible (e.g., Bernacchi et al. 2005; Hollinger et al. 2005). Using ecosystem‐scale flux measurements cannot resolve the total C present in an ecosystem; additional measurements of above‐and belowground biomass are needed to quantify C pools. However, eddy covariance fluxes can quantify whether C storage is increasing or decreasing over time without the signal‐to‐noise ratio challenges associated with direct measurements due to the continuous nature of the data collection.

In this opinion article, we synthesize published literature that represents a range of measurement techniques that directly address each of the three hypotheses presented in Figure 1. Significant data exist, as will be discussed in Section 2, to test hypothesis 1 (H1). We then use long‐term observed data (7–17 years) collected over several agricultural ecosystems located in the same climate region (Central Illinois, USA) over several years to test hypothesis 2 (H2)—that soil C achieves steady‐state under conventional tillage, as represented by a stable NECB, and hypothesis 3 (H3)—that management practices such as no‐till or perennial integration increase the net flux of C into agroecosystems. The data we present, as outlined below, have all been published previously but are integrated here to present the opinion that current assumptions of how agricultural ecosystem C dynamics have evolved over time based on management decisions might be flawed. Although our opinion is based on one location within the Midwest US, to our knowledge, this is the first synthesis of diverse agroecosystems monitored continuously over multiple years in the same climate using the gold‐standard eddy covariance method (Baldocchi 2020). Measuring all agroecosystems in the same general area removes the confounding influences presented by climate forcing and edaphic variability inherent in assimilating data from multiple locations. This work should be considered in the context of a single region, but we argue that it may be representative, at least in terms of directional responses in ecosystem C storage change, of similar cropping systems grown in other intensive agricultural production regions of the world.

## 2 Hypothesis 1: Significant Carbon Losses Arose From Land Conversion of Native Prairie to Annual Crops

Native mixed‐species prairie ecosystems with rich soil organic carbon (SOC) stocks dominated the landscape prior to the rapid expansion of row crop agriculture in the Midwest US. Before the invention of the steel plow by John Deere (Kendall 1959), Illinois is estimated to have had nearly 9 million hectares of prairie. Of this, less than 0.01% of the native prairie remains, primarily as remnants less than 4 ha in size. The disturbance of the soil profile from tillage practices reduces ecosystem C storage, as carbon dioxide is released by microbial respiration (David et al. 2009; DeLuca and Zabinski 2011). Decades of supporting empirical evidence show clear consensus that significant C losses occurred with the transition from prairie to cultivated crops (as reviewed by DeLuca and Zabinski 2011) and therefore, this effect has been integrated into ecosystem models (Matson et al. 1997; Parton et al. 2005; Voroney et al. 2011). From these well‐documented analyses, among others, there is evidence to support H1 (Figure 1), namely that significant ecosystem C losses occurred during the transition from perennial tallgrass prairie to conventional agricultural production in the Midwest US.

## 3 Hypothesis 2: After Initial Losses, C Steady‐State Is Achieved Under Conventional Till Cropping Systems

H2 postulates a steady‐state level of soil C will be achieved after the initial C loss from conversion of native prairie to row crops (Figure 1). Although C losses from soils associated with conventional tillage have been relatively well documented (reviewed in Mehra et al. 2018), the assumption of steady state is attributed to an increase in crop residue inputs to the soil that counteract soil C losses (e.g., Matson et al. 1997). This assumption has been widely accepted and integrated into measurement (e.g., Bernacchi et al. 2005) and model‐based scaling of changes in soil C (Mathers et al. 2023) and is being used to calculate payments in C trading platforms (Mishra et al. 2021).

**FIGURE 2:**
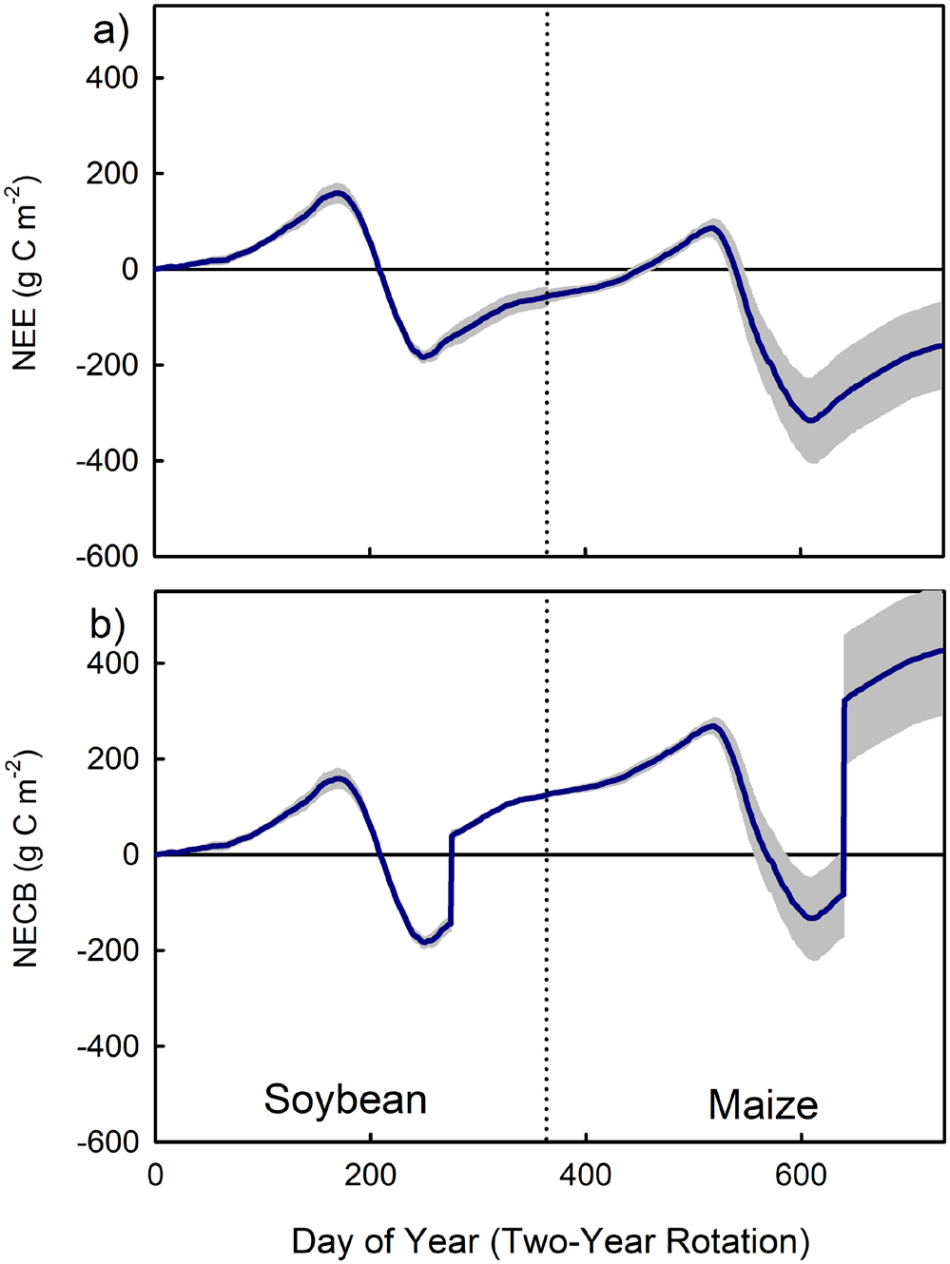
Mean time‐resolved cumulative net ecosystem exchange (a) and net ecosystem carbon balance (b) for a conventionally tilled soy‐maize system under cultivation for over a century. Data represents 6 years of maize data and 3 years of soybean data. The data are averaged over a 2‐year rotational cycle time period. Values below zero represent an annual net C uptake, whereas values above zero represent an annual net C loss from the ecosystem to the atmosphere. Shading represents the standard error of the mean of all years (3 annual cycles for soy and 6 annual cycles for maize). Figure produced using gap filled carbon flux data from eddy covariance systems as detailed in Moore et al. (2020) and Blakely et al. (2025).

Integrating long‐term flux measurements of NEE over time, we calculate net C exchange for a conventionally tilled maize‐soybean agroecosystem—representing the dominant land use and tillage practice for the majority of Midwest US agriculture. The data show the ecosystem represents an atmospheric C sink (Figure 2a), meaning the net flow of C between the atmosphere and ecosystem suggests C storage. The data in Figure 2, collected from a field that has been in continuous row crop agriculture for over a century, are the mean of several years of data reduced into a 2‐year soybean/corn rotational cycle, a typical agricultural practice in Midwestern row crop agriculture. A large portion of the atmospheric C sink in Figure 2a, however, is allocated to the corn and soybean grain, which is removed from the field but not accounted for in the NEE flux measurements. Once we account for the C in the harvested grain as a loss from the field, the ecosystem's NECB shows it becomes a net C source (Figure 2b).

Measuring C fluxes at the ecosystem scale captures the re‐emission of fixed C from microbial respiration of litter and belowground biomass back to the atmosphere. A well‐functioning soil microbiome is important for agroecosystem health and productivity (e.g., Berendsen et al. 2012; Zhang et al. 2017), with microbial activity (i.e., carbon respiration) and biomass turnover accelerated by conventional tillage practices (Nunes et al. 2020; Paustian et al. 2000). If timed with ideal climate and nutrient conditions, conventionally tilled agroecosystems can release substantial amounts of C back to the atmosphere (Abraha et al. 2018; Moore et al. 2022; Wang et al. 2020). These combined processes help explain the trends shown in Figure 2, which indicates steady‐state C conditions are not achieved under conventionally tilled cropping systems in the Midwest US, but they instead continue to lose C and are a net source of C to the atmosphere, refuting H2.

## 4 Hypothesis 3: Conservation Management Practices Reduce Disturbance and Increase Soil Organic Carbon

Given the extensive area that transitioned from prairie to row crop agriculture, the Midwest represents a critical region for implementing and monitoring land management strategies aimed at restoring C and increasing ecosystem C storage. H3 states that conservation management practices increase C storage in agricultural systems over time (Figure 1). For cropping systems specifically, this can include several practices, such as shifting from conventional tillage to no‐till practice, incorporating perennial crops into farming systems, and altering crop rotation regimes either through species combinations or cover cropping during fallow periods.

**FIGURE 3:**
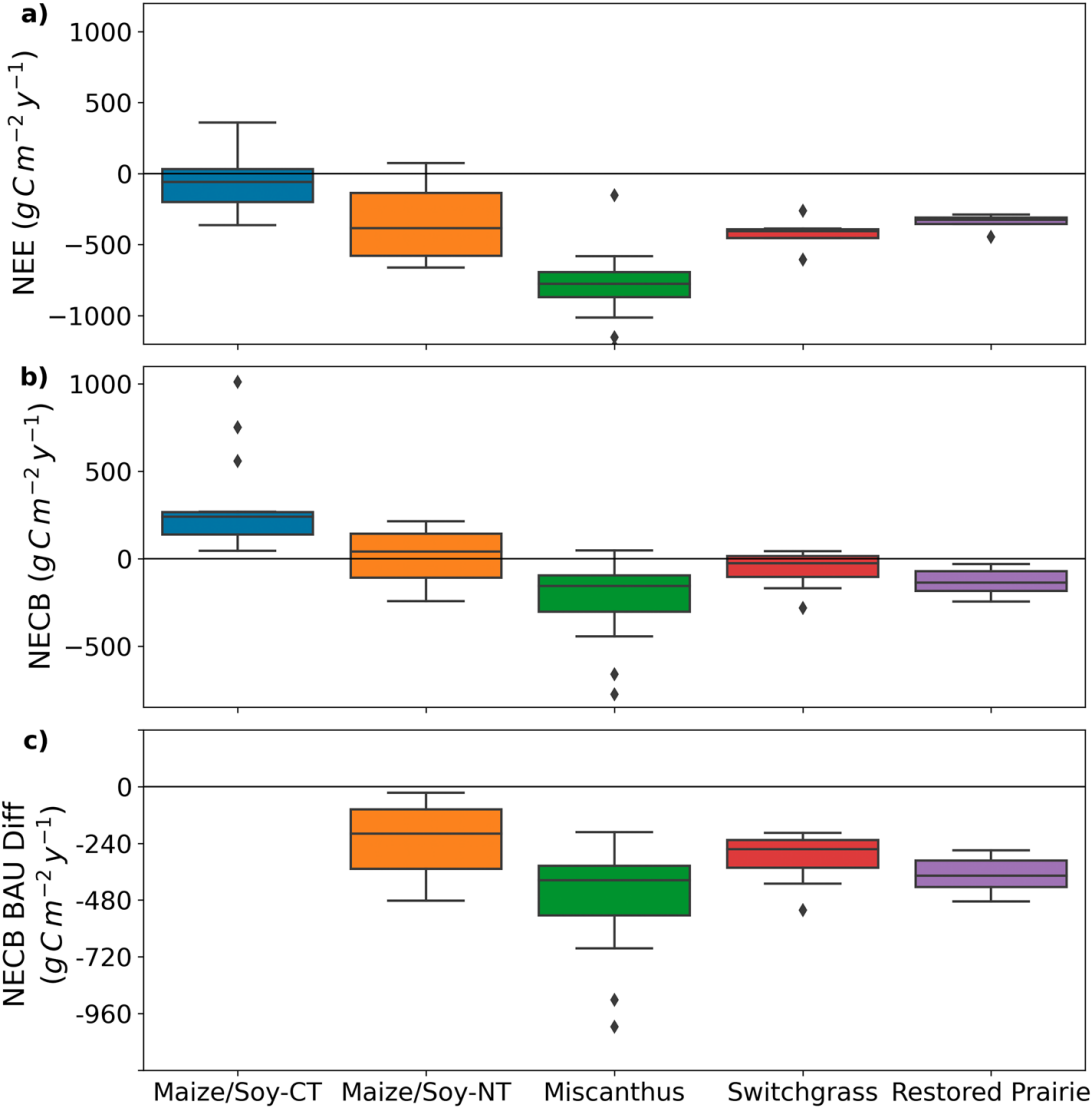
Average annual (a) net ecosystem exchange, (b) net ecosystem carbon budget (NECB) for different cropping systems in the Midwest US, and (c) the difference in NECB between that of the ‘business as usual’ (BAU) Maize/Soy‐CT agroecosystem to that of improved practice. Values are taken from previously published values (Bernacchi et al. 2005; Blakely et al. 2025; Moore et al. 2020; West et al. 2010) that are available from Ameriflix. The datasets specifically used were US‐UiA University of Illinois Switchgrass (Bernacchi et al. 2022a), US‐UiB University of Illinois Miscanthus (Bernacchi et al. 2023a), US‐UiC University of Illinois Maize‐Soy (Bernacchi et al. 2022b), US‐UiD University of Illinois Restored Native Prairie (Bernacchi et al. 2023b) and US‐Bo Bondville (Meyers 2016). Each dataset was quality assured and gap filled following the same method as outlined in Moore et al. (2020) for all sites. NECB was calculated using annual yield data based on dry mass for each crop and carbon content fraction of 0.48 for biomass harvests (Miscanthus, switchgrass and restored prairie) and 0.45 for grain harvests (maize‐soybean systems). CT refers to conventional tillage and NT refers to no‐till maize/soy systems. Number of years included for each system are included in parentheses as follows; maize/soy‐CT (14), maize/soy‐NT (17), miscanthus (14), switchgrass (7), and restored prairie (7). Values below zero represent an annual net uptake of carbon, whereas values above zero represent an annual net carbon loss from the system.

If we first consider no‐till cropping as a conservation management practice, evidence from around the world points to the success of this practice in building SOC, particularly in the 0–30 cm topsoil layers (Bohoussou et al. 2022; Cui et al. 2024; Mehra et al. 2018; Nunes et al. 2020; Ogle et al. 2019). This effect has been particularly noted in the Midwest US, where early indications from eddy covariance monitoring of no‐till practice showed a slight increase in ecosystem C sequestration compared to conventionally tilled systems (Bernacchi et al. 2005). However, after integrating more recent data into the prior analysis by Bernacchi et al. (2005), the long‐term mean NECB suggests that the no‐till maize‐soy system is net C neutral (Figure 3b), albeit still reducing C losses compared to conventionally tilled maize‐soy (Figure 3c).

Although evidence shows that no‐till maintains SOC, this may initially come at a cost to crop yield, particularly in humid, rainfed regions (Pittelkow et al. 2014). Further work has shown that C gains in the surface soils may be offset with losses at depth (> 20 cm) in no‐till systems (Ogle et al. 2019). Strategies to reduce these potential yield losses from implementing no‐till practice will be important to develop given the primary goal of agricultural systems—producing grain. Through our long‐term comparison of no‐till against conventional maize‐soy production in the Midwest US, we have found no evidence that conservation tillage in the form of no‐tillage increases ecosystem C storage, contradicting H3 (Figure 1). No‐till practices appear to stop C losses associated with conventional tillage, thus resulting in C steady‐state, but questions remain about how to keep the C in these systems in the long term while balancing economic, land management, and climate forcings. Furthermore, at least in the shorter term, no‐till may lead to an increase in N_2_O emissions (Six et al. 2004), which is important to consider when assessing the full GHG mitigation potential of various management practices (Robertson et al. 2000).

Another mechanism for storing C in ecosystems is to reduce the time a field spends in fallow by sowing cover crops that continue the capture of atmospheric C for soil storage after the primary crop is harvested. At our monitoring sites in the Midwest US, implementing cover crops for C storage purposes has not yet been investigated using ecosystem‐scale flux measurements. For a comprehensive analysis of the potential for C sequestration by cover crops implemented across US cropping systems, we direct readers elsewhere (Blanco‐Canqui and Blanco‐Canqui 2022; Jian et al. 2020; McClelland et al. 2021; Peng et al. 2023; Poeplau and Don 2015). These papers present a range of estimates for how croplands could realistically be used to store C through cover cropping. However, more long‐term datasets, such as those presented above, are necessary to understand the long‐term trends in C dynamics associated with implementing cover cropping (Yi et al. 2025). The long‐term outcomes of the ecosystem C balance for cover cropping systems thus remain subject to further investigation.

A third option for building ecosystem C, specifically SOC, involves the perennialization of agricultural land by either incorporating or switching to perennial crops. Perennial crops account for ~30% of global cropland area (Ledo et al. 2018), are planted without the need for annual replanting, and fall into two broad categories of woody or grassy species (Ledo et al. 2020). Without the need for annual replanting, perennial systems can build SOC over time through their longer growth periods, extensive below‐ground root networks, and aboveground litter inputs that are not periodically disturbed like annual ecosystems through tillage and other land management practices (Anderson‐Teixeira et al. 2013; Ledo et al. 2020; Robertson et al. 2017; Siddique et al. 2023). Additional benefits of perennial crops for mitigating GHG emissions often require less fertilizer inputs to produce biomass (Heaton et al. 2009; Ruan et al. 2016; Smith et al. 2013) and have demonstrated resilience in growing in land areas where annual crop yield is poor (Edmonds et al. 2021; Gelfand et al. 2013) and GHG emissions are relatively high (Lawrence et al. 2021).

Over several years of measurement in the Midwest US, we found that two major grassy perennial cropping systems (*Miscanthus* × *giganteus*; miscanthus and *Panicum virgatum* ; switchgrass), as well as a perennial restored prairie, were net sinks of C even after harvest, as evidenced by their negative NECB (Figure 3). This finding is in contrast with their conventionally tilled annual crop counterparts (i.e., maize and soybean), which have a positive NECB and are a net C source to the atmosphere (Figures 2 and 3). These perennial crops began building ecosystem C soon into their establishment (Anderson‐Teixeira et al. 2013; Zeri et al. 2011)—a finding that applies to perennial crops more broadly (Ledo et al. 2020). However, if land is converted from native or restored systems (i.e., Conservation Reserve Program lands), the C payback time for perennial crops can still be long and may extend beyond the lifecycle of the crop itself (Abraha et al. 2019; Blanc‐Betes et al. 2023; Fargione et al. 2008; Gelfand et al. 2011). Provided careful land selection for crop establishment is adhered to and incorporating perennial crops does not shift GHG emissions elsewhere, our long‐term observations show perennial cropping systems effectively increase C storage in agricultural systems.

## 5 Once There, How Do We Keep the Carbon in the System?

An important consideration for any farming system looking to build SOC, as well as the C crediting market more generally, is how to maintain C storage in ecosystems (see Oldfield et al. 2022). As discussed in Section 4, some conservation or perennialization management practices may lead to ecosystem C storage, but this can be quickly reversed if management practices change or GHG emissions are transferred elsewhere. For example, if market pressures lead a grower to revert a perennial system back to a conventionally tilled cropping system, the stored C built up by the perennial system can be quickly respired back to the atmosphere (Abraha et al. 2019; Gelfand et al. 2011; Moore et al. 2020). Increased occurrence of extreme and unseasonal climate events can also create conditions that accelerate SOC loss from soils (Black et al. 2017; Castellano et al. 2011; von Haden et al. 2019), or prevent sustained rates of C accumulation (Frank et al. 2015). If extreme climate events are coupled with changes in management, this can further accelerate the loss of C from agricultural soils (e.g., Moore et al. 2022). There are also certain agricultural production regions where conservation management practices are more successful than others at storing SOC, either due to climate, soil type, or socioeconomic constraints (Lessmann et al. 2022; Ogle et al. 2019). Addressing the unresolved issues of regional equality and SOC longevity in emerging carbon markets will be important for ensuring the ongoing success of such markets in effectively reducing agricultural GHG emissions.

## 6 Uncertainties, Assumptions and Opportunities

While our analysis is representative of one location within the Midwest US cropping region, it demonstrates the importance of long‐term data collection over agricultural systems to properly quantify and monitor C emissions and storage in agricultural systems. The Midwest US is a significant contributor to annual global crop production (USDA‐FAS 2024). Reporting the long‐term annual variability in C fluxes and the soil C pools that they influence in these systems with in‐field observed data provides important insight for other regions of the world looking to trade in agricultural C credits. We also acknowledge that our analysis did not account for the additional role of tillage practice on other factors that can affect C losses, including wind and water erosion of the soil that have been shown to accelerate C losses from conventionally tilled systems (Thaler et al. 2022). We also did not factor in the role of other GHGs, such as nitrous oxide and methane, on regulating the overall C balance of cropping systems on an annual basis. Although methane emissions are typically low in rain‐fed systems like the Midwest US (Omonode et al. 2007), this may not be the same for all agricultural regions of the world. The rate of nitrous oxide emission from agricultural ecosystems can be vastly different depending on factors, such as soil type, climate, fertilizer application rate, and plant uptake capacity (Decock 2014; Hoben et al. 2011; Lawrence et al. 2021; Yang et al. 2022), and can negate SOC storage, leading to an overestimation of net climate benefit (Lugato et al. 2018). As such, efforts to increase ecosystem C storage should carefully consider the overall global warming potential implications of management decisions (Lawrence et al. 2021).

Another important limitation is introduced by the eddy covariance technique itself. Although this technique represents the current best practice for ecosystem scale measurements, no measurement technique is perfect and is subject to experimental error (Baldocchi 2014). Additional information is also needed to correctly interpret eddy covariance data and calculate net C balance in agricultural systems, which for cropping systems includes annual field‐specific yield information at minimum. Despite these technical constraints, the eddy covariance technique is still the best way of collecting high temporal resolution ecosystem‐scale measurements of C fluxes and indicative change in SOC pools over time in agricultural systems. Historically, the technique has mainly been applied for research purposes and long‐term (i.e., > 10 year) studies in agricultural systems have been limited (e.g., Baldocchi 2020). The advancement of global research infrastructures, including eddy covariance flux towers for C monitoring (Beringer et al. 2022; Huber et al. 2021; Novick et al. 2018), coincides with a growing need to account for C emissions in the agricultural sector (e.g., Mitchell et al. 2024; Oldfield et al. 2024) and the expansion of nature‐based climate solutions as a mechanism for lowering emissions (e.g., Novick et al. 2024). Together, this creates an opportunity to more accurately monitor C cycle variability. Long‐term measurements of C dynamics and targeted research design, as presented by our long‐term analysis, can advance scientific understanding of C cycle components for various agronomic ecosystems. More investment in long‐term in situ measurements is therefore critical to better understand how variation in growing environment might alter the potential for C storage in agroecosystems. Alternatively, the development of affordable, low‐maintenance sensor suites to measure ecosystem C fluxes can enable replicated experimental designs using ecosystem C flux measurements that lead to rigorous pair‐wise statistical analyses, potentially accomplishing the same goal without the need for decade or longer continuous monitoring.

## 7 Conclusion

With numerous publications documenting C losses from the transition of native prairie to row crop agriculture and over 50 site years of field‐scale C flux measurements from several cropping systems, we now have the data required to begin to assess long‐term C losses and gains in high production Midwest US agroecosystems. These measurements demonstrate the long‐lasting C cost of land conversion for maize and soybean production (H1) and the continued loss of C from conventionally tilled systems. Our results suggest that the assumptions of steady‐state and increases in SOC storage over time (H2) may not hold for all agricultural ecosystems and in all locations. With conservation tillage practices (i.e., no‐till) C losses can be reduced compared to conventional tilled systems. However, it is with the incorporation of perennial crops in farming systems that our results indicate ecosystem C storage can occur. Even with the C storage benefits of perennials (H3), keeping the C in these systems under variable climate, management, and socioeconomic pressures will remain a challenge for the future. Our findings demonstrate the potential for agricultural ecosystems to store C while challenging the widely accepted, and perhaps incorrect, assumptions that conventionally tilled agroecosystems have achieved steady‐state C dynamics and that conservation tillage practices increase C storage. The data synthesized in this article clearly demonstrate that conservation practices can reduce C losses associated with conventional tillage.

## Author Contributions

**Caitlin E. Moore:** conceptualization, data curation, formal analysis, investigation, methodology, software, validation, visualization, writing – original draft. **Bethany Blakely:** data curation, formal analysis, investigation, methodology, software, validation, writing – review and editing. **Taylor L. Pederson:** data curation, investigation, software, validation, writing – review and editing. **Nuria Gomez‐Casanovas:** investigation, validation, writing – review and editing. **Christy D. Gibson:** validation, writing – review and editing. **Anya M. Knecht:** project administration, validation, writing – review and editing. **Guler Aslan‐Sungur:** validation, writing – review and editing. **Evan H. DeLucia:** funding acquisition, methodology, project administration, resources, supervision, writing – review and editing. **Emily A. Heaton:** validation, writing – review and editing. **Andy VanLoocke:** validation, writing – review and editing. **Tilden Meyers:** funding acquisition, methodology, project administration, resources, supervision, writing – review and editing. **Carl J. Bernacchi:** conceptualization, data curation, formal analysis, funding acquisition, investigation, methodology, project administration, resources, supervision, validation, visualization, writing – original draft.

## Conflicts of Interest

The authors declare no conflicts of interest.

## Data Availability

The data that support the findings of this study are openly available from Dryad at http://doi.org/10.5061/dryad.kd51c5bjt. AmeriFlux was obtained from https://doi.org/10.17190/AMF/1617725, https://doi.org/10.17190/AMF/1846665, https://doi.org/10.17190/AMF/1846664, https://doi.org/10.17190/AMF/1987605, and https://doi.org/10.17190/AMF/1246036.
